# Supplementary material for: Genetic and environmental influence on alcohol intent and alcohol sips among U.S. children–Effects across sex, race, and ethnicity
Source: PLoS One. 2024 Feb 15;19(2):e0298456. doi: 10.1371/journal.pone.0298456 (PMC10868864; doi:10.1371/journal.pone.0298456)
Supplement: S1 Appendix — (DOCX) [file pone.0298456.s001.docx]

**Genetic and Environmental Influence on Alcohol Intent and Alcohol Sips among U.S. Children – Effects across Sex, Race, and Ethnicity**

Troy Puga^1,2^ BS, Yadi Liu^1^ BS, Peng Xiao^3^ PhD, Ran Dai^1^ PhD, Hongying Daisy Dai^1^ PhD

^1^ College of Public Health, University of Nebraska Medical Center. Omaha, NE, USA.

^2^ College of Osteopathic Medicine, Kansas City University. Kanas City, MO, USA.

^3^ Dept. of Genetics, Cell Biology & Anatomy, University of Nebraska Medical Center. Omaha, NE, USA.

**Supplementary Information**

**Table of Contents:**

Supplementary Material 1. Measures, code book, and analytical scripts.

Supplementary eTable1. Original variables defined in ABCD.

Supplementary Material 2. Comprehensive Analysis using ACE, AE, and CE models.

Supplementary eTable 2. Variance Component Analysis of Genetic Heritability for Alcohol-Related Traits

Supplementary eTable 3. Estimates of genetic and environmental variance components for susceptibility (intent) of alcohol use, overall and stratified by gender and race.

Supplementary eTable 4. Estimates of genetic and environmental variance components for initiation of alcohol use, overall and stratified by gender and race.

**Supplementary Material 1. Measures, code book, and analytical scripts.**

**Supplementary eTable1. Original variables defined in ABCD.**

| Variable: Question | Condition | Responses | Source |
| --- | --- | --- | --- |
| Substance Use | | | |
| tlfb_alc: Have you heard of ____ alcohol, such as beer, wine or liquor? | None | 0 = No; 1 = Yes | Youth Survey |
| tlfb_alc_sip: I want to start by asking if you have EVER TRIED any of the following drugs in your life. Have you ever tried___________at any time in your life? A sip of alcohol such as beer, wine or liquor (rum, vodka, gin, whiskey) | tlfb_alc == 1 | 0 = No; 1 = Yes | Youth Survey |
| path_alc_youth2: Have you ever been curious about drinking alcohol? | tlfb_alc ==1 && tlfb_alc_sip ==0 | 1 = Very curious;  2 = Somewhat curious;  3 = A little curious;  4 = Not at all curious;  5 = Don't know;  6 = Refused to answer | Youth Survey |
| Demographics | | | |
| interview_age: Age in months at the time of the interview/test/sampling/imaging | Age is rounded to chronological month. If the research participant is 15-days-old at time of interview, the appropriate value would be 0 months. If the participant is 16-days-old, the value would be 1 month. | 0 :: 1260 | Youth Interview |
| Sex: Sex of subject at birth |  | M = Male;  F = Female; O=Other;  NR = Not reported | Parent PhenX Demographics Survey |
| race_ethnicity: Race Ethnicity (Child) |  | 1 = White;  2 = Black;  3 = Hispanic;  4 = Asian;  5 = Other | Parent PhenX Demographics Survey |
| genetic_zygosity_status_1: Genetically inferred zygosity status between participant and genetic_paired_subjectid_1 |  | 1= monozygotic ; 2= dizygotic ; 3=siblings ;  -1= not available (twins/sibs, genetic_pi_hat not calculated) | Youth Biospecimens: Blood (DNA) |

- Alcohol (susceptibility) was defined using the following code:

if (path_alc_youth2=**1** | path_alc_youth2=**2** | path_alc_youth2=**3**) | (path_alc_youth5=**1** | path_alc_youth5=**2** | path_alc_youth5=**3**) | (path_alc_youth8=**1** | path_alc_youth8=**2** | path_alc_youth8=**3**) then susp_alc=**2**; /*Yes*/

if path_alc_youth2=**4** & path_alc_youth5=**4** & path_alc_youth8=**4** then susp_alc=**1**; /*No*/

- Alcohol sip (initiation) was defined using the following code:

if tlfb_alc=**0** then alc_ever=**1**; /*No*/

if tlfb_alc=**1** & tlfb_alc_sip=**0** then alc_ever=**1**; /*No*/

if tlfb_alc=**1** & tlfb_alc_sip=**1** then alc_ever=**2**; /*Yes*/

- The code book can be accessed at <https://nda.nih.gov/data_structure.html?short_name=abcd_ysu02>

<https://nda.nih.gov/data_structure.html?short_name=acspsw03>

- Data can be accessed through NIMH Data Archive (NDA) (https://nda.nih.gov/abcd).

**Supplementary Material 2. Comprehensive Analysis using ACE, AE, and CE models.**

**Supplementary eTable 2. Variance Component Analysis of Genetic Heritability for Alcohol-Related Traits**

|  | **Alcohol Susceptibility** | **Alcohol Initiation** |
| --- | --- | --- |
| **Pearson Correlation (p-value)** |  |  |
| Dizygotic Twins | r=0.05  (p=0.37) | r=0.43  (p<.0001) |
| Monozygotic Twins | r=0.19  (p=0.007) | r=0.44  (p<.0001) |
| **ACE model*** |  |  |
| Initial Value | S1=0.5, S2=0.5 | S1=0.5, S2=0.5 |
| BIC | 1338.0 | 1883.7 |
| Additive Genes (*h^2^* ) | 0.31 (-0.28 to 0.91)  P=0.3023 | 0.04 (-0.30 to 0.37)  p=0.83 |
| Common Environment (*c^2^*) | 0 | 0.62 (0.37 to 0.86)  P<.0001 |
| Unique Environment (*e^2^*) | 0.69 (0.45 to 0.93)  P<.0001 | 0.35 (0.22 to 0.47)  P<.0001 |
| **CE model*** |  |  |
| Initial Value | S1=0.5, S3=0.5 | S1=0.5, S3=0.5 |
| BIC | 1333.2 | 1877.0 |
| Common Environment (*c^2^*) | 0.20 (0.033 to 0.37)  P=0.019 | 0.64 (0.57 to 0.72)  P<.0001 |
| Unique Environment (*e^2^*) | 0.80 (0.63 to 0.97)  P<.0001 | 0.36(0.28 to 0.43)  P<.0001 |
| **AE model*** |  |  |
| Initial Value | S1=0.5, S3=0.5 | S1=0.5, S3=0.5 |
| BIC | 1331.8 | 1683.2 |
| A^2^ (Heritability  from Additive Genes) | 0.28 (0.084 to 0.49)  P=0.0055 | 0.98(0.97 to 0.98)  P<.0001 |
| E^2^  (Unique Environment) | 0.72 (0.52 to 0.92)  P<.0001 | 0.024(0.018 to 0.030)  P<.0001 |

**Supplementary eTable 3. Estimates of genetic and environmental variance components for susceptibility (intent) of alcohol use, overall and stratified by gender and race.**

|  | **Overall** | **Male** | **Female** | **White** | **Black** | **Hispanics** |
| --- | --- | --- | --- | --- | --- | --- |
| **Correlation** |  |  |  |  |  |  |
| DZ | r=0.05  p=0.37 | r=0.02  p=0.8 | r=0.07  p=0.39 | r=0.09  p=0.25 | r=-0.08  p=0.57 | r=0.01  p=0.95 |
| MZ | r=0.19  p=0.007 | r=0.22  p=0.03 | r=0.15  p=0.16 | r=0.06  p=0.50 | r=0.32  p=0.11 | r=0.44  p=0.03 |
| **ACE model*** |  |  |  |  |  |  |
| Initial Value | S1=0.5, S2=0.5 | S1=0.5, S2=0.5 | S1=0.5, S2=0.5 | S1=0.5, S2=0.5 | S1=0.5, S2=0.5 | S1=0.5, S2=0.5 |
| BIC | 1338.0 | 99.2 | 77.9 | 222.1 | 65.4 | 62.1 |
| Additive Genes (*h^2^* ) | 0.31 (-0.28 to 0.91)  P=0.3023 | 0.38  (0.31 to 0.44)  p<.0001 | 0.39  (0.067 to 0.71)  p=0.02 | 0.34  (0.29 to 0.40)  p<.0001 | 0.48  (0.44 to 0.52)  p<.0001 | 0.53  (0.39 to 0.67) p<.0001 |
| Common Environment (*c^2^*) | 0 | 0 | 0 | 0 | 0 | 0 |
| Unique Environment (*e^2^*) | 0.69 (0.45 to 0.93)  P<.0001 | 0.62  (0.56 to 0.69)  p<.0001 | 0.61  (0.29 to 0.93)  P=0.0002 | 0.66  (0.60 to 0.71)  p<.0001 | 0.52  (0.48 to 0.56)  p<.0001 | 0.47  (0.33 to 0.61) p<.0001 |
| **CE model*** |  |  |  |  |  |  |
| Initial Value | S1=0.5, S3=0.5 | S1=0.5, S3=0.5 | S1=0.5, S3=0.5 | S1=0.5, S3=0.5 | S1=0.5, S3=0.5 | S1=0.5, S3=0.5 |
| BIC | 1333.2 | 707.6 | 631.9 | 870.7 | 197.8 | 159.0 |
| Common Environment (*c^2^*) | 0.20 (0.033 to 0.37)  P=0.019 | 0.24(0.0030 to 0.47)  P=0.0472 | 0.20(-0.052 to 0.45)  P=0.1194 | 0.16(-0.060 to 0.38)  P=0.1541 | 0.14(-0.30 to 0.58)  P=0.5365 | 0.28(-0.19 to 0.75)  P=0.2359 |
| Unique Environment (*e^2^*) | 0.80 (0.63 to 0.97)  P<.0001 | 0.76(0.53 to 1.00)  P<.0001 | 0.80(0.55 to 1.05)  P<.0001 | 0.84(0.62 to 1.01)  P<.0001 | 0.86(0.42 to 1.30)  P=0.0002 | 0.72(0.26 to 1.19)  P=0.0029 |
| **AE model*** |  |  |  |  |  |  |
| Initial Value | S1=0.5, S3=0.5 | S1=0.5, S3=0.5 | S1=0.5, S3=0.5 | S1=0.5, S3=0.5 | S1=0.5, S3=0.5 | S1=0.5, S3=0.5 |
| BIC | 1331.8 | 710.4 | 637.7 | 877.6 | 201.9 | 162.8 |
| A^2^ (Heritability  from Additive Genes) | 0.28 (0.084 to 0.49)  P=0.0055 | 0.25 (-0.0057 to 0.50)  P=0.0554 | 0.26 (-0.040 to 0.56)  P=0.0894 | 0.15 (-0.11 to 0.40)  P=0.2535 | 0.28 (-0.23 to 0.79)  P=0.2753 | 0.37 (-0.14 to 0.89)  P=0.1528 |
| E^2^  (Unique Environment) | 0.72 (0.52 to 0.92)  P<.0001 | 0.75 (0.50 to 1.00)  P<.0001 | 0.74 (0.44 to 1.04)  P<.0001 | 0.85 (0.60 to 1.10)  P<.0001 | 0.72 (0.21 to 1.22)  P=0.0057 | 0.63 (0.11 to 1.14)  P=0.0180 |

**Supplementary eTable 4. Estimates of genetic and environmental variance components for initiation of alcohol use (alcohol sip), overall and stratified by gender and race.**

|  | **Overall** | **Male** | **Female** | **White** | **Black** | **Hispanics** |
| --- | --- | --- | --- | --- | --- | --- |
| **Prevalence (% yes)** |  |  |  |  |  |  |
| All individuals | 25.40 | 28.46 | 22.20 | 28.66 | 8.13 | 23.96 |
| Dizygotic Pairs | 25.09 | 28.04 | 22.14 | 29.25 | 4.43 | 22.03 |
| Monozygotic Pairs | 25.86 | 29.08 | 22.29 | 27.77 | 14.77 | 27.03 |
| **Correlation** |  |  |  |  |  |  |
| DZ | r=0.44  p<.0001 | r=0.42  p<.0001 | r=0.44  p<.0001 | r=0.41  p<.0001 | r=-0.05  p=0.69 | r=0.51  p<.0001 |
| MZ | r=0.44  p<.0001 | r=0.43  p<.0001 | r=0.43  <.0001 | r=0.38  p<.0001 | r=0.55  p=0.0001 | r=0.66  p<.0001 |
| **ACE model** |  |  |  |  |  |  |
| Initial Value | S1=0.5, S2=0.5 | S1=0.5,  S2=0.5 | S1=0.5, S2=0.5 | S1=0.5, S2=0.5 | S1=0.5, S2=0.5 | S1=0.5, S2=0.5 |
| BIC | 1883.7 | 1035.4 | 876.8 | 1328.0 | 147.5 | 202.9 |
| Additive Genes (*h^2^* ) | 0.04 (-0.30 to 0.37)  p=0.83 | 0.09  (-0.38 to 0.56)  p=0.70 | 0.03  (-0.50 to 0.55)  p=0.91 | 0 | 0.54  (-0.16 to 1.24)  p=0.13 | 0.32  (-0.20 to 0.84)  p=0.23 |
| Common Environment (*c^2^*) | 0.62 (0.37 to 0.86)  P<.0001 | 0.55  (0.19 to 0.91)  p=0.003 | 0.63  (0.25 to 1.01)  p=0.001 | 0.60  (0.50 to 0.70)  p<.0001 | 0.21  (-0.73 to 1.15)  p=0.66 | 0.54  ( 0.077 to 1.00)  p=0.02 |
| Unique Environment (*e^2^*) | 0.35 (0.22 to 0.47)  P<.0001 | 0.36  (0.19 to 0.52)  p<.0001 | 0.34  (0.14 to 0.54)  p=0.0007 | 0.40  (0.30 to 0.50)  p<.0001 | 0.25  (-0.19 to 0.69)  p= 0.27 | 0.14  (-0.052 to 0.34)  p=0.15 |
| **CE model*** |  |  |  |  |  |  |
| Initial Value | S1=0.5, S3=0.5 | S1=0.5, S3=0.5 | S1=0.5, S3=0.5 | S1=0.5, S3=0.5 | S1=0.5, S3=0.5 | S1=0.5, S3=0.5 |
| BIC | 1877.0 | 1029.4 | 870.7 | 1321.7 | 144.5 | 199.0 |
| Common Environment (*c^2^*) | 0.64 (0.57 to 0.72)  P<.0001 | 0.62(0.51 to 0.73)  P<.0001 | 0.65(0.54 to 0.76)  P<.0001 | 0.60(0.50 to 0.70)  P<.0001 | 0.60(0.32 to 0.87)  P<.0001 | 0.75(0.59 to 0.92)  P<.0001 |
| Unique Environment (*e^2^*) | 0.36(0.28 to 0.43)  P<.0001 | 0.38(0.27 to 0.49)  P<.0001 | 0.35(0.24 to 0.46)  P<.0001 | 0.40(0.30 to 0.50)  P<.0001 | 0.40(0.13 to 0.68)  P=0.0041 | 0.25(0.083 to 0.42)  P=0.0038 |
| **AE model*** |  |  |  |  |  |  |
| Initial Value | S1=0.5, S3=0.5 | S1=0.5, S3=0.5 | S1=0.5, S3=0.5 | S1=0.5, S3=0.5 | S1=0.5, S3=0.5 | S1=0.5, S3=0.5 |
| BIC | 1683.2 | 1058.3 | 895.8 | 1359.0 | 149.8 | 170.3 |
| A^2^ (Heritability  from Additive Genes) | 0.98(0.97 to 0.98)  P<.0001 | 0.58 (0.43 to 0.72)  P<.0001 | 0.62 (0.50 to 0.75)  P<.0001 | 0.55 (0.43 to 0.68)  P<.0001 | 0.55 (0.26 to 0.83)  P=0.0003 | 0.98 (0.97 to 1.00)  <.0001 |
| E^2^  (Unique Environment) | 0.024(0.018 to 0.030)  P<.0001 | 0.42 (0.28 to 0.57)  P<.0001 | 0.38 (0.25 to 0.50)  P<.0001 | 0.45 (0.32 to 0.57)  P<.0001 | 0.45 (0.17 to 0.74)  P=0.0022 | 0.02 (0.0019 to 0.033)  P=0.0287 |
